# Fecal calprotectin level correlated with both endoscopic severity and disease extent in ulcerative colitis

**DOI:** 10.1186/s12876-016-0462-z

**Published:** 2016-04-12

**Authors:** Kousaku Kawashima, Shunji Ishihara, Takafumi Yuki, Nobuhiko Fukuba, Naoki Oshima, Hideaki Kazumori, Hiroki Sonoyama, Noritsugu Yamashita, Yasumasa Tada, Ryusaku Kusunoki, Akihiko Oka, Yoshiyuki Mishima, Ichiro Moriyama, Yoshikazu Kinoshita

**Affiliations:** Department of Internal Medicine II, Shimane University Faculty of Medicine, 89-1, Enya-cho, Izumo, Shimane 693-8501 Japan; Division of Internal Medicine, Matsue Seikyo General Hospital, 8-8-8, Nishitsuda, Matsue, 690-8522 Japan; Cancer center, Shimane University Hospital, 89-1, Enya-cho, Izumo, Shimane 693-8501 Japan

**Keywords:** Ulcerative colitis, Fecal calprotectin, Disease extent

## Abstract

**Background:**

The relationship between fecal calprotectin (FC) and disease extent in ulcerative colitis (UC) has not been fully elucidated. The aim of this study was to clarify the correlation of FC with disease extent and severity in UC patients.

**Methods:**

UC patients scheduled to undergo an ileocolonoscopy were enrolled and fecal samples for FC measurement were collected prior to the procedure. A Mayo endoscopic subscore (MES) was determined for each of 5 colonic segments. To evaluate the association of FC with extent of affected mucosa as well as disease severity, we assessed the correlation of FC level with the sum of MES (S-MES) for the 5 colonic segments as compared to the maximum score of MES (M-MES).

**Results:**

FC measurements in conjunction with findings from 136 complete colonoscopies in 102 UC patients were evaluated. FC level showed a stronger correlation with S-MES (correlation coefficient *r* = 0.86, *p* < 0.001) as compared to M-MES (*r* = 0.79, *p* < 0.001). In patients with an M-MES of 1, 2, and 3, FC level showed a significant correlation with S-MES (*r* = 0.67, *p* < 0.001; *r* = 0.70, *p* < 0.001; *r* = 0.47, *p* = 0.04, respectively). Our findings indicate that FC level is elevated in patients with greater areas of affected mucosa even in those with the same M-MES value.

**Conclusions:**

FC level was shown to be correlated with the extent of affected mucosa as well as severity in UC patients, thus it is useful for precise assessment of mucosal inflammation.

## Background

Ulcerative colitis (UC) is a chronic idiopathic disorder characterized by mucosal inflammation in the colon and rectum. In recent years, management of UC has substantially changed, with newly introduced treatments such as biologics and tacrolimus shown to result in significant endoscopic improvement as well as clinical remission in some cases [1–8]. Therefore, endoscopic mucosal healing (MH), which is associated with sustained clinical remission and reduced rates of hospitalization and surgical resection, has emerged as a major treatment goal for UC patients [9–11]. Although an endoscopy is recognized as the most reliable method for evaluating MH, that examination is relatively invasive and sometimes painful, while a colonoscopy procedure may exacerbate UC [12]. As a result, it is difficult to perform frequent endoscopic assessments of affected mucosa in clinical practice and alternative noninvasive methods are necessary for assessment of mucosal inflammation associated with UC.

Calprotectin is a 36-kDa calcium- and zinc-binding protein that represents most of the cytosolic proteins of granulocytes [13]. Measurement of fecal calprotectin (FC), which is stable for up to 3 days at room temperature and resistant to degradation [14], is useful, as its level reflects migration of neutrophils through the inflamed bowel wall to the mucosa and has been widely utilized to clarify its correlation with endoscopically proven UC activity [15–21]. Based on previously reported data showing a strong correlation with endoscopic activity, noninvasive measurement of FC level has been proposed to provide a reliable surrogate marker of mucosal inflammation associated with UC [22].

Numerous scoring systems have been developed for assessment of endoscopic activity in UC patients, of which the Mayo endoscopic subscore (MES) is one of the most widely employed endoscopic indices [23]. MES is divided into 4 points (0–3) based on proctosigmoiddoscopic appearance. When an ileocolonoscopy is performed, MES is often regarded as the maximum score for the parts of the colorectum examined. In addition, most other endoscopic indices also use such a maximum score for assessment of colonic mucosal activity [24–27]. In previous reports of the correlation of FC with mucosal activity, the endoscopic scores used were those obtained for the most severe level of inflammation in the colorectum and disease extent was not considered to be related to UC for evaluating the clinical usefulness of FC. In this regard, the correlation of FC with disease extent in UC has not been fully investigated.

In the present study, we assessed the sum of MES (S-MES) for the 5 colonic segments in patients with UC who underwent a complete colonoscopy. To clarify the correlation of FC with extent of affected mucosa as well as disease severity, we investigated the correlation of FC level with S-MES as compared to the maximum score of MES (M-MES).

## Methods

This study was prospectively conducted from February 2013 to December 2014 at Shimane University Hospital and Matsue Seikyo General Hospital in Japan. The protocol was approved by the ethical committees of both hospitals and all patients gave written informed consent, and the study was performed in adherence to the Helsinki Declaration.

### Patients

Patients with a previously established diagnosis of UC and scheduled to undergo an ileocolonoscopy were enrolled. For those who were outpatients, we asked them to obtain fecal samples within 7 days before the ileocolonoscopy, which were brought to the hospital or sent by postal mail immediately after collection. Fecal samples from inpatients were obtained the day before the ileocolonoscopy and examined in the hospital laboratory. Patients who previously underwent a sigmoidoscopy, or with a history of colorectal surgery, acute infectious enterocolitis, or regular intake of aspirin and/or other nonsteroidal anti-inflammatory drugs (NSAIDs) were excluded from this study. Those unable to provide a fecal sample were also excluded. When exacerbation of symptoms such as diarrhea and melena were noted prior to the ileocolonoscopy examination, we performed a stool culture for diagnosis of pathogenic bacteria infection including *Clostridium difficile*, as well as serum and histological examinations to exclude CMV infection, as necessary. For patient demographics, age, sex, duration of disease, clinical activity, extent type of UC, and concomitant medications being taken at each colonoscopic examination were noted. The extent type of UC was determined according to the Montreal classification based on previous colonoscopic findings, unless the extent of affected mucosa based on the present colonoscopic findings revealed more extensive involvement [28, 29].

### Fecal calprotectin measurement

Collected fecal samples used for FC measurements were stored at −20 °C until shipment to the laboratory (SRL Inc., Tokyo, Japan), where the FC level was determined with the examiner blinded to the endoscopic findings using a quantitative enzyme-linked immunosorbent assay (PhiCal®, Immundiagnostik, Germany), with a standard method. The detection limits of this ELISA kit for FC range from 5.3 to 2100 μg/g.

### Colonoscopic findings and clinical disease activity

Patients with UC received a magnesium citrate- or polyethylene glycol-based electrolyte solution for bowel preparation prior to the ileocolonoscopy. All of the procedures (EVIS 260 series, Olympus, Tokyo, Japan) were performed by 2 of the authors (K.K. and T.Y.). Patients who underwent an incomplete colonoscopy examination (cecum not reached) were excluded, as some with UC had maximum inflammation in the right colon [30]. Colonoscopic findings were finally determined according to the MES for each of the 5 portions of the colorectum (cecum to ascending colon, transverse, descending, sigmoid colon, rectum). Those determinations were made by the first 5 authors of this study (K.K., S.I., T.Y., N.F., N.O.), each an experienced gastroenterologist, within 1 week after the colonoscopy. When endoscopic grading differed among them, the final grade was decided based on consensus. We precisely defined the maximum score of MES as M-MES, while the sum of MES for the 5 colonic segments, which ranged from 0 to 15 was determined as S-MES. Clinical disease activity was evaluated on the day of the ileocolonoscopy using Rachmilewitz’ clinical activity index (CAI) consisting of 7 subscores, as previously reported [27]. Clinical remission was defined as a CAI value of 4 or less.

### Statistical analysis

Statistical analysis was performed using the SPSS statistical package (version 19.0, SPSS, Chicago, USA). Parametric numerical results are presented as the mean ± standard deviation (SD), while nonparametric data are presented as the median and interquartile range (IQR). A Mann-Whitney test was used to investigate differences between nonparametric data. Correlation analyses were performed using Spearman’s rank correlation test. All *P*-values are two-sided and *P* <0.05 was considered to be statistically significant.

## Results

### Patients

The clinical characteristics of the present UC patients are shown in Table 1. A total of 136 complete colonoscopies that were accompanied by fecal sample examinations were performed in 102 UC patients. Of those, 32 (24 %) were performed in patients with clinical activity (CAI ≥5), while the other 104 (76 %) were performed in those with clinical remission (CAI ≤4). Of the 136 colonoscopies, 68 (50.0 %) were performed for pancolitis, 36 (26.4 %) for left-sided colitis, and 32 (23.6 %) for proctitis.

**Table 1:** Characteristics of study patients and colonoscopic findings

Patients	
Total	102
Gender	
Male	65 (64 %)
Female	47 (36 %)
Number of colonoscopy	
Once	71 (70 %)
Twice	28 (27 %)
3 times	3 (3 %)
Complete colonoscopy	
Total number	136
Median age in years (IQR)	44.5 (31.0-63.5)
Median duration of disease in years (IQR)	7.0 (4.0-12.5)
Clinical activity	
Remission	104 (76 %)
Active	32 (24 %)
Purpose of colonoscopy	
Evaluation of disease	46 (34 %)
Surveillance	90 (66 %)
Concomitant medications	
Aminosalicylate	129 (95 %)
Topical aminosalicylate	35 (26 %)
Corticosteroids	23 (17 %)
Topical steroids	8 (6 %)
Azathioprine/Mercaptopurine	37 (27 %)
Tacrolimus	11 (8 %)
Biologics	13 (10 %)
Colonoscopic findings (maximum score in colorectum)	
Mayo endoscopic subscore 0	35 (26 %)
Mayo endoscopic subscore 1	33 (24 %)
Mayo endoscopic subscore 2	47 (35 %)
Mayo endoscopic subscore 3	21 (15 %)
Biochemical Results	
Median C-reactive protein (mg/l) (IQR)	0.05 (0.03-0.12)
Median serum albumin (g/l) (IQR)	4.4 (4.0-4.6)
Median white blood cell count (10^3^×/μL) (IQR)	5.88 (4.86-7.52)
Median hemoglobin (g/dL) (IQR)	13.8 (12.2-14.9)
Median platelet count (10^4^×/μL) (IQR)	24.7 (20.8-29.3)

*IQR* interquartile range

### Correlation of FC level with both M-MES and S-MES

**Fig. 1:**
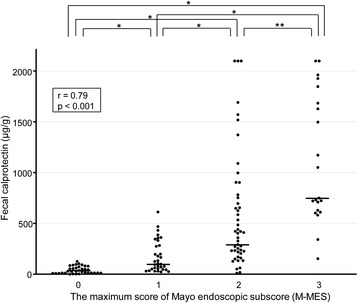
Scatterplot showing correlation of fecal calprotectin (FC) level with maximum of Mayo endoscopic subscore (M-MES). The median and interquartile range (IQR) for FC levels in patients with an M-MES of 0 (*n* = 35), 1 (*n* = 33), 2 (*n* = 47), and 3 (*n* = 21) were 35.2 (17.3-76.6), 103.3 (55.2-336.4), 295.0 (162.9-1000.0), and 751.9 (632.8-1685.6) μg/g, respectively. *p < 0.001, **p < 0.01

**Fig. 2:**
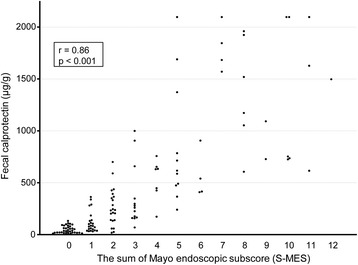
Scatterplot showing correlation of fecal calprotectin (FC) level with sum of Mayo endoscopic subscore (S-MES) for 5 colonic segments

**Fig. 3:**
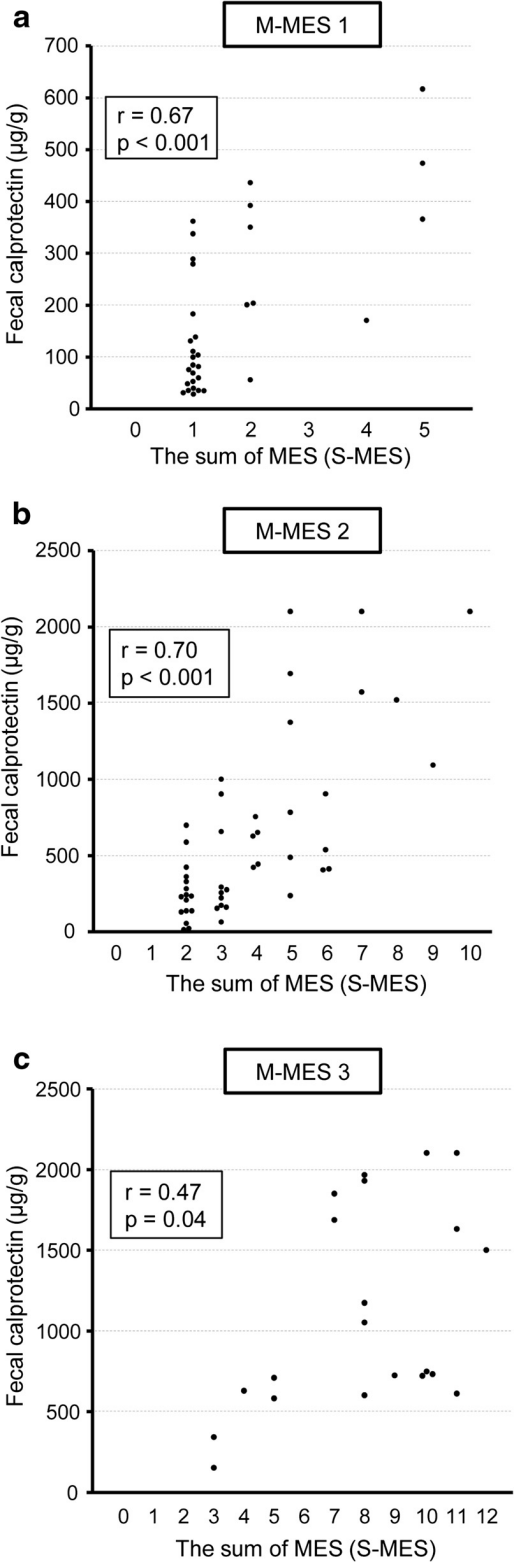
Scatterplot showing correlation of fecal calprotectin (FC) level with sum of Mayo endoscopic subscore for 5 colonic segments (S-MES) in patients with a maximum MES (M-MES) of 1, 2, and 3. **a** M-MES 1. **b** M-MES 2. **c** M-MES 3

As shown in Fig. 1, the median FC level in patients with an M-MES of 0 (*n* = 35), 1 (*n* = 33), 2 (*n* = 47), and 3 (*n* = 21) was 35.2 (IQR 17.3-76.6), 103.3 (55.2-336.4), 295.0 (162.9-1000.0), and 751.9 (632.8-1685.6) μg/g, respectively. FC was significantly elevated in accordance with endoscopic severity and showed a significant correlation with M-MES (Spearman’s rank correlation coefficient *r* = 0.79; *p* < 0.001). Furthermore, FC demonstrated a strong correlation with S-MES (*r* = 0.86, *p* < 0.001), as shown in Fig. 2. In patients with an M-MES of 1, 2, and 3, FC demonstrated a significant correlation with S-MES (*r* = 0.67, *p* < 0.001; *r* = 0.70, *p* < 0.001; *r* = 0.47, *p* = 0.04, respectively), as shown in Fig. 3a–c. These results indicated that FC level is correlated with the extent of affected mucosa even in patients with the same M-MES value as well as endoscopic severity.

### FC levels in patients with the same S-MES, but different extent of affected mucosa

We also compared FC levels in patients with the same S-MES, but different extent of affected mucosa. Of 22 with an S-MES of 2, an MES of 1 in 2 segments (S-MES = 1 + 1 = 2 points) was seen in 6 and an MES of 2 in only 1 segment (S-MES = 2 points) was seen in 16, with FC level in the former at 272.2 ± 144.0 μg/g and at 256.7 ± 192.2 μg/g in the latter, not a significant difference (*p* = 0.86).

## Discussion

An important element of the present study is that MES was determined in each of the 5 colonic segments to evaluate the extent of affected mucosa as well as endoscopic severity. Our results clearly showed that FC level is significantly correlated with both endoscopic severity and the extent of affected mucosa in patients with UC, revealing the importance of its measurement as an indicator of mucosal inflammation.

FC level has been widely examined in regard to its correlation with endoscopic activity in UC patients, with correlation coefficients between FC level and UC endoscopic activity reported to range from 0.51 to 0.83 [15–21]. In those studies, the endoscopic index for UC was determined by examining the part of the colorectum with the most severe inflammation. Similarly, in the present study, we evaluated mucosal severity in UC patients using M-MES, and found that the correlation coefficient between FC level and M-MES was 0.79, which correlated well with those previous reports. Thus, our findings confirmed that FC level is correlated with endoscopic severity in the most severely affected segment of the colorectum.

In most previous reports, the UC endoscopic index was determined using the most severely affected segment in the colorectum [23–27]. However, segmental colonic assessment is becoming increasingly important in cases of UC according to the United States Food and Drug Administration [30, 31]. Furthermore, the recent proposed definition of mucosal healing by the International Organization for the study of Inflammatory Bowel Disease includes absence of friability, blood, erosions, and ulcers in all gastrointestinal mucosa segments [31]. Another report also noted that the recently developed UC colonoscopic index of severity (UCCIS) is relevant as an endoscopic index, because of its scoring system, which evaluates all colonic segments [32]. Thus, endoscopic assessment of all segments of the colorectum is recommended for investigating disease extent as well as severity in UC patients.

To investigate the correlation between FC level and extent of affected mucosa, we used the sum of MES (S-MES) in all 5 colonic segments in the preset study, as MES is the most widely used endoscopic index for UC and considered to be highly reliable. Very recently, Lobaton T, et al. proposed a modified Mayo endoscopic score as a new endoscopic index for UC, which is calculated based on analysis of the MES for each of the 5 colonic segments [33]. In our study, the correlation coefficient value for the correlation of FC with S-MES was 0.86, nearly as high as the value (0.73) reported in their study. Furthermore, we found that FC level had a stronger correlation with S-MES (*r* = 0.86) as compared to M-MES (*r* = 0.79), also similar to that previous study. Based on these results, we concluded that FC level likely has a stronger correlation with the sum of MES for the 5 colonic segments as compared to the maximum score of MES.

A novel finding presented in our study that differentiates it from other previous reports is clarification of the significant correlation of FC level with S-MES, even in UC patients with the same M-MES value (Fig. 3). Our results also indicate that FC is elevated in conjunction with a greater area of affected mucosa even in UC patients with the same classification of endoscopic severity. Therefore, we concluded that FC level in UC patients is important for precise assessment of mucosal inflammation by showing the extent of affected mucosa as well as severity. In addition, the correlation coefficient of FC with S-MES in patients with an M-MES of 3 was comparatively low at 0.47 as compared to those in patients with an M-MES of 1 or 2, though those cannot be directly compared. FC level in patients with an M-MES of 3 is higher due to excessive mucosal severity, while the influence of the extent of affected mucosa on FC level might be relatively low in those patients.

In addition, we compared FC levels in patients with the same S-MES, but different extent of affected mucosa. The result suggests that FC level is similar between patients with mild activity in a greater extent of mucosa and those with moderate activity in a limited extent. However, to clearly show this point, examinations of a larger number of enrolled subjects will be necessary.

Our study has some limitations. First, it was conducted at 2 different hospitals in Japan. To reduce inter-observer variations as much as possible, before performing the FC measurement, all colonoscopic findings were finally determined by 5 experienced gastroenterologists, each of whom had at least 10 years of experience with performing colonoscopic examinations. However, additional investigations that include a greater number of institutions will be necessary. Second, our cohort included few UC patients with an M-MES of 3, as some of those had difficulty with providing an adequate fecal sample due to lack of consistency. In addition, an incomplete colonoscopy may easily occur in UC patients with an M-MES of 3 due to their severe condition and pain related to the procedure. Finally, we did not perform histological examinations of colonic biopsy samples. Further investigations based on histological examination findings obtained from all 5 colonic segments including endoscopically non-inflamed mucosa are necessary for understanding the correlations of those results with endoscopic findings and FC level.

## Conclusions

The present results revealed that FC level is significantly correlated with the extent of affected mucosa as well as disease severity in patients with UC, and its measurement is important for precise assessment of mucosal inflammation.

## Abbreviations

CAI: clinical activity index
FC: fecal calprotectin
IQR: interquartile range
MES: Mayo endoscopic subscore
MH: mucosal healing
M-MES: maximum score of Mayo endoscopic subscore
S-MES: sum of Mayo endoscopic subscore
UC: Ulcerative colitis
